# Vulnerable Jugendliche in sich verändernden Risikoumgebungen: Figurationen von urbaner Jugendwohnungslosigkeit in Deutschland, Dänemark und Großbritannien

**DOI:** 10.1007/s12592-021-00387-0

**Published:** 2021-10-20

**Authors:** Frank Sowa, Kristian Fahnøe, Alastair Roy, Alan Farrier, Marco Heinrich, Mette Kronbæk

**Affiliations:** 1 Fakultät Sozialwissenschaften, Technische Hochschule Nürnberg Georg Simon Ohm, 90121 Nürnberg, Deutschland; 2 Department of Social Work, Faculty of Social Education, Social Work and Administration, University College Copenhagen, 2000 Frederiksberg, Dänemark; 3 Psychosocial Research Unit, School of Social Work, Care and Community, Faculty of Health and Care, University of Central Lancashire, PR1 2HE Preston, Großbritannien; 4 Faculty of Health and Care, Brook Building, Healthy and Sustainable Settings Unit, School of Community Health and Midwifery, PR1 2HE Preston, Großbritannien

**Keywords:** Jugendwohnungslosigkeit, Risikoumgebungen, Wohlfahrtsstaatliche Politik, Soziale Arbeit, Lebenswelt, Youth homelessness, Risk environments, Welfare state policies, Social services, Lifeworld

## Abstract

Dieses Projekt stellt die Frage, wie die COVID-19-Pandemie Prozesse der sozialen Inklusion und Exklusion von wohnungslosen Jugendlichen beeinflusst. Anhand von drei internationalen Fallstudien soll das Verständnis dafür verbessert werden, wie Pandemien die Risiken und die zugänglichen wohlfahrtsstaatlichen Ressourcen für junge Menschen mit Wohnungslosigkeitserfahrung prägen. Das Wissen, das in dieser vergleichenden Studie entwickelt wird, kann dazu beitragen, die Art und Weise zu verbessern, wie Organisationen der Wohnungslosenhilfe auf neue, veränderte oder verstärkte Probleme und Bedürfnisse von jungen wohnungslosen Menschen reagieren.

## Abstract (en)

This project asks how the COVID 19 (coronavirus disease 2019) pandemic has affected processes of social exclusion and inclusion for homeless young people. It will advance the understanding of how pandemics shape the risks and accessible welfare resources for young people experiencing homelessness through three international case studies. The knowledge developed in this comparative study will help to improve how social service organizations respond to new or intensified needs of young homeless people.

## Problemstellung und Forschungsstand

In mehreren Ländern wurde dokumentiert, dass wohnungslose Menschen infolge der Entwicklungen im Rahmen der COVID-19-Pandemie besonderen Risiken ausgesetzt sind (z. B. durch Schließungen von wohlfahrtstaatlichen und sozialarbeiterischen Hilfeeinrichtungen oder durch Stigmatisierungen der Betroffenen), und dass wohnungslose Menschen infolge der gesellschaftlichen Reaktionen auf die Pandemie (z. B. „lockdowns“ oder „social distancing“) mit extremeren Problemen konfrontiert sind (Kirby 2020). Viele wohlfahrtsstaatliche Hilfeeinrichtungen mussten als Reaktion auf die Pandemie ihre Konzepte und Angebotsformen ändern und versuchen, Unterstützung in Zeiten anzubieten, in denen sowohl Mitarbeiter*innen als auch Nutzer*innen mit neuen Risiken und veränderten Ressourcen umgehen müssen. Junge wohnungslose Menschen leben in einem komplexen Beziehungsgeflecht, das als Figurationen der Jugendwohnungslosigkeit beschrieben werden kann, welche als Muster der gegenseitigen Abhängigkeit zwischen Gruppen verstanden werden (Elias 1997). Sie müssen ihr eigenes Überleben sichern, indem sie Bewältigungsstrategien, strategisches und taktisches Expert*innenwissen über das Leben auf der Straße sowie strategische Allianzen mit Organisationen der Sozialen Arbeit und sozialpolitischen Institutionen entwickeln (Roy 2016). Die COVID-19-Pandemie hat dazu geführt, dass vulnerable Jugendliche mit modifizierten sozialen, physischen und ökonomischen Umständen bzw. neuen Risikoumgebungen konfrontiert sind (Rhodes 2002). Diese Veränderungen werden zum Teil durch politische Reaktionen auf die Pandemie beeinflusst, von denen viele explizit auf Organisationen der Sozialen Arbeit abzielen. Die Erfahrungen von wohnungslosen Jugendlichen werden jedoch auch von den Wohlfahrtspolitiken beeinflusst, die die Risiken, denen die Jugendlichen ausgesetzt sind, verstärkt oder verringert haben.

## Forschungsfragen

Das internationale, von der VolkswagenStiftung finanzierte Forschungsprojekt untersucht die Auswirkungen der COVID-19-Pandemie auf junge, von Wohnungslosigkeit betroffene Menschen sowie auf sozialarbeiterische Hilfeeinrichtungen in den drei Wohlfahrtsstaaten Deutschland, Großbritannien und Dänemark. Dies geschieht durch drei zusammenhängende Forschungsfragen:

- *Makroebene:* Wie hat die Pandemie die Figurationen wohnungsloser junger Menschen in den Wohlfahrtssystemen in Deutschland, Dänemark und Großbritannien verändert?
- *Mesoebene:* Wie haben die Wohlfahrtsverbände und Hilfeeinrichtungen in Deutschland, Dänemark und Großbritannien auf die Pandemie reagiert, um die Unterstützung für wohnungslose junge Menschen aufrechtzuerhalten?
- *Mikroebene:* Wie hat die Pandemie die aktuellen Risiken und Bedürfnisse, die wohnungslose junge Menschen in ihrem täglichen Leben erfahren, und ihre Bewältigungsstrategien verändert?

## Theoretischer und konzeptioneller Rahmen

Jennifer Rankin und Sue Regan (2004, S. 11) weisen darauf hin, dass, je komplexer die Bedürfnisse einer Person sind, es desto wahrscheinlicher wird, dass sie durch die Lücken der von der Gesellschaft bereitgestellten Dienste fällt. Die Lücken in den sozialen Diensten verändern sich zweifelsohne durch die COVID-19-Pandemie. Eine Möglichkeit, die komplexen sozialen Situationen, mit denen wohnungslose junge Menschen konfrontiert sind, zu begreifen, ist der „risk environment“-Ansatz (Rhodes 2002), bei dem Risiken und Schäden auf Umweltfaktoren zurückzuführen sind und diese verstärken oder reduzieren (Duff 2010). Im Kontext dieses Projekts zielt der Ansatz darauf ab, den Fokus für Veränderungen von den Individuen allein auf die sozialen Situationen und Strukturen zu verlagern, in denen sich die Individuen befinden. Dies hilft dabei, die Verflechtung der gesellschaftlichen Reaktion auf COVID-19 auf der Makroebene (z. B. Politiken, die den Zugang zu öffentlichem Raum einschränken), der Mesoebene (z. B. die Reorganisation von Hilfsangeboten) und der Mikroebene (z. B. das Alltagsleben junger Menschen, die in Wohnungslosigkeit leben) zu verstehen. Der Ansatz eignet sich insbesondere, um die komplexen Veränderungen auf der *gesellschaftlichen Ebene*, die durch COVID-19 hervorgerufen wurden, die *physischen Bewegungen* (z. B. Zugang zu öffentlichen Räumen und Verkehrsmitteln), die *sozialen Interaktionen* (z. B. aufgrund sozialer Distanzierungsregeln) und *die ökonomischen Umstände* (z. B. eingeschränkte Möglichkeiten zum Betteln), die die soziale Situation und die Bewältigungsstrategien der Menschen beeinflussen, zu erforschen und vor diesem Hintergrund die veränderte Situation junger wohnungsloser Menschen zu rekonstruieren.

Aufgrund der ländervergleichenden Anlage der Studie wird der Risikoumgebungsansatz durch Gøsta Esping-Andersens (1990) Typologie von Wohlfahrtsstaatsregimen ergänzt. Dadurch können die sich verändernden Wohlfahrtspolitiken in drei nationalen Kontexten berücksichtigt und nachvollzogen werden. Die wohlfahrtsstaatlichen Politiken bilden die sozialpolitische Hintergrundfolie für die professionellen, personenbezogenen Dienstleistungen der Sozialen Arbeit. Die komplexen Herausforderungen der Sozialen Arbeit mit jungen wohnungslosen Menschen in verschiedenen wohlfahrtsstaatlichen Kontexten als Reaktion auf die Pandemie werden ebenso im Fokus des Projekts stehen wie die Zusammenhänge zwischen politischen Maßnahmen und den Risiken und Ressourcen derjenigen jungen Menschen, die bereits am Rande der Gesellschaft stehen.

## Forschungsdesign und methodisches Vorgehen

Im Zentrum des Forschungsprojekts stehen drei qualitative Fallstudien in den drei unterschiedlichen Wohlfahrtsstaaten Deutschland (konservatives Regime), Dänemark (sozialdemokratisches Regime) und Großbritannien (liberales Regime) (Esping-Andersen 1990). Den Ausgangspunkt der Untersuchung bildet in jedem Land eine Organisation der Problembearbeitung, die Dienstleistungen für junge Menschen, die in Wohnungslosigkeit leben, anbietet. Dieser länderübergreifende und regionale Ansatz wird Unterschiede und Gemeinsamkeiten zwischen spezifischen nationalen und regionalen politischen Reaktionen auf COVID-19 in verschiedenen Wohlfahrtsregimen und die Auswirkungen auf der Ebene der Praxis und der gelebten Erfahrung identifizieren.

Zur Beantwortung der Forschungsfragen wird ein qualitatives Forschungsdesign gewählt. Auf der *Mikroebene* steht die Analyse der Bewältigungsstrategien, Erfahrungen und Wahrnehmungen junger wohnungsloser Menschen in sich verändernden Risikoumgebungen im Vordergrund. Hierfür werden in den Partnerorganisationen acht bis zehn junge Menschen zweimal interviewt. Das erste qualitative Interview dient der Erfassung der Biografie und Lebenssituation (Back 2007). Nach dem Interview werden die Jugendlichen ermutigt, Veränderungen in ihrem Leben und ihrem Alltag schriftlich oder visuell in einem Tagebuch („Eine Woche im Leben von …“) festzuhalten, beispielsweise wohin sie gehen, wen sie sehen, wo sie übernachten, was sie essen, wie sie Unterstützung erhalten. Das zweite Interview findet eine Woche später statt und wird die derzeitige Alltagssituation thematisieren. Die Tagebuchaufzeichnungen bilden dabei eine Grundlage für die Erzählungen. Das übergeordnete Ziel ist die Generierung von fokussierten Erzählungen über das tägliche Leben und die Lebenswelten der Informant*innen. Je nach den geltenden Einschränkungen durch die Pandemie werden die Interviews persönlich, per Telefon oder online durchgeführt.

Auf der *Mesoebene* werden die Auswirkungen der Pandemie auf Praktiken untersucht, um die Herausforderungen und Antworten der Sozialen Dienste, insbesondere in Form neuer Konzepte und Formen der Unterstützung, in der Ära der Pandemie zu erheben. Dabei wird analysiert, mit wem Sozialarbeiter*innen arbeiten und wie sie arbeiten (physische, telefonische und Online-Räume). Die Expert*innen-Interviews werden Auskunft darüber geben, wie die Pandemie die Praxislandschaft verändert hat und wie die Hilfeeinrichtungen in der Lage waren, die Unterstützung für junge wohnungslose Menschen aufrechtzuerhalten. Es werden vier lokale Praktiker*innen aus jeder Fallstudienorganisation rekrutiert, mit denen leitfadengestützte Interviews geführt werden. Gegenstand dieser Interviews werden das alltägliche Arbeiten (Dake und Roy 2018) und die Interaktionen mit den Adressat*innen von Sozialer Arbeit sein.

Schließlich erfolgt auf der *Makroebene* eine Analyse der Rahmensetzung durch Wohlfahrtsregime, um zu verstehen, wie die Wohlfahrtsstaaten Deutschland, Dänemark und Großbritannien auf die Pandemie reagiert haben. Bewältigungsstrategien von wohnungslosen Individuen und lokale Reaktionen von Organisationen der Sozialarbeit hängen von den nationalen, regionalen und lokalen Kontextbedingungen ab. Diese ermöglichen oder schränken Handeln ein. Daher werden lokale (kommunal)politische Dokumente, Folgenabschätzungen, Aktionspläne, Protokolle und Verfahren, die als Reaktion auf die Pandemie erstellt wurden, in eine Dokumentenanalyse mit einbezogen und Auswirkungen auf der Mikro- und Mesoebene identifiziert.

Der multimethodische und mehrere Ebenen umfassende Zugang führt zu drei Fallstudien in einem nationalen Wohlfahrts- und Politikkontext, die im internationalen Vergleich analysiert werden, um ein breiteres Verständnis der Reaktionen von jungen, vulnerablen Menschen und Wohlfahrtsorganisationen auf die COVID-19-Pandemie zu erzielen. Aus diesen Ergebnissen werden Empfehlungen für Politik und Praxis entwickelt. Die Schlüsselbotschaften der Forschung werden für verschiedene Zielgruppen aufbereitet. So ist u. a. die Zusammenarbeit mit dem Drehbuchautor und Trickfilmzeichner Jamie Munro (www.mistermunro.co.uk) vorgesehen, der einen kurzen Animationsfilm produzieren wird. Auf diese Weise wird es möglich sein, neue Figurationen urbaner Jugendwohnungslosigkeit in Deutschland, Dänemark und Großbritannien zu visualisieren und über die neuen Risikoumgebungen von vulnerablen Jugendlichen aufzuklären.
